# Epistemic injustice in the therapeutic relationship in psychiatry

**DOI:** 10.1007/s11017-023-09627-1

**Published:** 2023-05-24

**Authors:** Eisuke Sakakibara

**Affiliations:** Department of Neuropsychiatry, the University of Tokyo Hospital, Tokyo, Japan

**Keywords:** Doctor–patient relationship, Epistemic injustice, Objectification, Professional, Psychiatry

## Abstract

The notion of epistemic injustice was first applied to cases of discrimination against women and people of color but has since come to refer to wider issues related to social justice. This paper applies the concept of epistemic injustice to problems in the therapeutic relationship between psychiatrists and psychiatric patients. To this end, it is necessary to acknowledge psychiatrists as professionals with expertise in treating mental disorders, which impair the patient’s rationality, sometimes leading to false beliefs, such as delusions. This paper classifies the characteristic features of the therapeutic relationship in psychiatry into three stages: those of a professional–client relationship, those of a doctor–patient relationship, and those of a psychiatrist–psychiatric patient relationship. Epistemic injustice is prevalent in psychiatric care owing to prejudice against patients with mental disorders. However, it is also predisposed by the roles that psychiatrists play in relation to psychiatric patients. This paper suggests some ameliorative measures based on the analysis.

## Introduction

In her book *Epistemic injustice: Power and the ethics of knowing*, Miranda Fricker [1] proposed the notion of a specifically epistemic kind of injustice. She indicated that there are two kinds of epistemic injustice: testimonial and hermeneutical injustice. Testimonial injustice refers to cases in which a hearer unjustly deflates the credibility of a speaker due to identity prejudice against the speaker’s social identity, such as with women and people of color. Hermeneutical injustice refers to situations in which people with particular social attributes have been excluded for such a long time from epistemic activities in a society that they are deprived of the conceptual resources to give meaning and voice to their experiences. Researchers have since pointed out that epistemic injustice can take many forms besides those identified by Fricker, such as testimonial smothering [2] and participant injustice [3].

While Fricker’s [1] book focused on the epistemic injustice associated with discrimination against women and people of color, recently, the concept of epistemic injustice has been applied to problems in a variety of other domains, including education [4], the judicial system [5], and healthcare [6]. This paper investigates the epistemic injustice suffered by people with mental disorders in psychiatric care.^1^ However, since this is a broad topic, further specification of the problem is required.

Now, a distinction should be made between, first, the epistemic injustice that occurs in the relationship between psychiatric patients and mental health professionals within the therapeutic relationship and, second, the epistemic injustice that occurs when patients with relatively stable mental health conditions are involved in or excluded from psychiatric research, the process of developing guidelines for psychiatric treatment, or the operation of mental healthcare systems. Anke Bueter, for example, stated that psychiatric patients have not been allowed to express their views in the process of developing diagnostic criteria for mental disorders, which she identified as a preemptive form of testimonial injustice [7]. This paper, however, concentrates on issues of epistemic injustice that arise in the relationship between psychiatric patients and mental health professionals during patients’ treatment.

Havi Carel and James Kidd [6, 8] noted that patients are likely to suffer from epistemic injustice in the healthcare system. Furthermore, along with Paul Crichton [9], they showed that people with mental disorders are even more vulnerable to epistemic injustice than those with physical disorders. For example, Elyn Saks [10] wrote about an experience in which she visited an emergency room due to an unusual and severe headache; however, the emergency doctor did not offer a proper examination because she had been diagnosed with schizophrenia. As a result, the subarachnoid hemorrhage she was suffering from was overlooked. Conversely, Spencer and Carel [11] found that positive stereotypes of mental disorders, such as obsessive–compulsive disorder (OCD) patients being intelligent and high-functioning, can lead to the trivialization and wrongful depathologization of OCD, constituting epistemic injustice to OCD patients. Furthermore, A.P. Scrutton [12] and A.J.M. Tate [13] criticized the status quo in psychiatry — where the medical model is considered as the sole valid model for explaining abnormal experiences such as hearing voices — thereby devaluing the plurality of perspectives and epistemically alienating people who have such experiences.

Fricker’s [1] original argument emphasizes the role of identity prejudice as a cause of epistemic injustice. This paper argues that the epistemic injustice experienced by patients with mental disorders in therapeutic relationships does not solely stem from prejudice against them, but also is predisposed by the roles that mental health professionals play in their relationships with patients. Such epistemic injustice is not caused by mere bad luck or the professional’s ill will but is associated with the very aspiration of the professional who is committed to work for the patient.

Accordingly, this paper focuses on the relationship between psychiatric patients and psychiatrists, in which epistemic injustice typically occurs, although it can also occur between psychiatric patients and other types of mental health professionals. In order to focus on the problem of epistemic injustice stemming from the roles played by psychiatrists, it is necessary to articulate the particularities of the therapeutic relationship between psychiatrists and patients with mental disorders. This paper classifies the characteristic features of the relationship into three stages: of a professional–client relationship, of a doctor–patient relationship, and of a psychiatrist–psychiatric patient relationship. These three relationships have a nested structure. A doctor–patient relationship is a kind of professional–client relationship, and a psychiatrist–psychiatric patient relationship is a kind of doctor–patient relationship.^2^

The rest of the paper proceeds as follows. In the "Epistemic injustice in the professional–client relationship" section, it is argued that in a professional–client relationship, professionals define the scope of their expertise and are responsible for ensuring the truthfulness of various matters within that scope. Next, the "Epistemic injustice in the doctor–patient relationship" section shows that in a doctor–patient relationship, the doctor is in charge of treating the patient’s illness and places a high priority on objective findings rather than subjective complaints. Then, the "Epistemic injustice in the psychiatrist–psychiatric patient relationship" section considers epistemic injustice in the psychiatrist–psychiatric patient relationship, in which the psychiatrist is sometimes required to regard some of the patient’s utterances as symptoms of their illness. These sections show that patients with mental disorders are prone to suffer from various forms of epistemic injustice in their relationship with their psychiatrists. Finally, the "Ameliorating epistemic injustice in therapeutic relationships in psychiatry" section presents recommendations to ameliorate epistemic injustice in the therapeutic relationship in psychiatry.

## Epistemic injustice in the professional–client relationship

### Characteristic features of a professional–client relationship

A professional (henceforth, denoted as “she” for convenience) is an expert who helps a client (“he” unless obviously otherwise), usually a layperson, for a fee. Doctors are one category of professionals. In a society with advanced division of labor, people in occupations are experts in the domains related to their work and earn their living based on their expertise. However, not all occupations are regarded as professions.

Wilbert Moore’s [14] classic analysis identified five factors that characterize professions. The first factor is occupation, which distinguishes a professional from an amateur. Making a living by providing a service is a necessary condition for a person to be considered as a professional, albeit not a sufficient condition.

The second factor is calling. Professions require commitment from those who practice them. Professionals are expected to choose their work of their own will and to devote their lives to it. It is considered inappropriate to hold a second job or use a profession as a temporary step toward another job.

The third factor is institution. Professions form professional associations, which integrate the common interests of professionals to encourage the government to implement policies in their favor. Professional associations also control the issuance and withdrawal of licenses to maintain service levels.

The fourth factor is education. Professionals must be educated. A professional must acquire abstract and theoretical knowledge through university education in addition to an apprenticeship or on-the-job training.

The fifth factor is service orientation. Professionals have a work ethic that dictates that they use their knowledge and skills to benefit their clients. Any behavior for personal gain that may harm clients is considered unprofessional and would be severely criticized by peers, sometimes leading to license revocation.

Moore [13] considered the boundary between professional and non-professional occupations to be ambiguous, the spectrum ranging from such non-professional occupations as carpenters to such typical professions as doctors and lawyers, depending on the five aforementioned factors. Moore counted engineers, university professors, scientists, and military professionals among professionals; but in this paper, by “profession,” I imply specifically the so-called helping professions. These include psychologists, pharmacists, accountants, financial planners, and social workers, in addition to the classic examples of doctors, lawyers, and clergy.

It might be objected that, since carpenters earn a fee for helping their customers, what is the difference between carpentry and helping professions? In response, carpenters are paid to make or repair things using their skills. They communicate with their customers only to the extent necessary to know what their customers want. By contrast, the core part of the task of helping professionals is *consultation*. Professionals listen to clients’ complaints and provide advice. In other words, professionals help clients solve problems by “renting” their mind — equipped with their expertise and experience — as an extension of clients’ minds [15]. This is one of the reasons that academic education is essential for a profession.

On the other side, a client who consults a professional is a layperson who typically lacks expert knowledge in a particular field. A client consults a professional for matters that he rarely experiences and that are difficult to deal with on his own. So, on the one hand, the client has first-person experience of his problem but does not have the concepts to formulate or the ideas to solve the concern. A professional, on the other hand, might not have personal experience with the problem. However, she has acquired a “formula” for understanding and solving the problem based on her second-person experience of working on many similar cases and third-person knowledge learned from the literature. Professionals apply pre-acquired formulas to the problems that their clients bring to them. In the medical profession, the “formula” would be a diagnosis of illness and its standard treatment. For professionals to make a living by solving clients’ problems, they must define the areas they deal with and conceptualize the complaints brought by clients based on the pre-existing formula.

When a client consults a professional, he must disclose confidential information or reveal his most private aspects to the professional that he might not ordinarily share with others. For this to be possible, the client must trust the professional and believe that she will not misuse the information or divulge the same to others. Conversely, the professional must trust that the client is not concealing important information from her, and that the client will act according to mutually agreed upon action plans. The professional’s success in solving the client’s problems is predicated on mutual trust and good epistemic teamwork between the two parties.

### Unwarranted credibility judgments

Epistemic injustice impedes epistemic teamwork between professionals and clients, increasing the likelihood of failure in the consultation process. This subsection examines unwarranted credibility judgments as instances of epistemic injustice that can arise in the professional–client relationship.

The professional–client relationship is characterized by the clients’ asymmetry of knowledge in the professional’s area of expertise. Based on this asymmetry, a professional often denies the client’s testimony. For example, assume that a patient with schizophrenia, whose condition has been stabilized by antipsychotic medication, sees an article on the Internet advertising that a special herbal formulation will cure schizophrenia and obviate the need for medication. If he confides in his psychiatrist that he would like to discontinue his medication and try the herbal formulation, the psychiatrist has to firmly advise the patient that such treatment lacks evidence and that the risks of relapse will increase if he discontinues antipsychotic medication. The point is that professionals must critically examine the reports and opinions of their clients in the areas in which they specialize; indeed, the client often expects such critical reviews.

This does not mean that the professional unfairly underestimates the credibility of their client. When what the client claims is inconsistent with standard knowledge in the professional’s specialty or with the material brought by the client (e.g., pay stubs, contract documents, and/or reports of laboratory tests), it is reasonable to infer that there must be something wrong with the client’s testimony.

Accordingly, testimonial injustice arises when a professional casts doubt on a client’s claim by devaluing their credibility based on prejudice against their social identity, such as being a woman or belonging to a lower socio-economic class. However, since this issue is not unique to professional–client relationships, this paper does not discuss it further.

Another unwarranted credibility judgment that can arise in the professional–client relationship includes the client’s overestimation of the credibility of the professional. Elianna Fetterolf [16] has affirmed the existence of socioculturally stipulated credibility stereotypes. Some stereotypes are negative, while others are positive. Undoubtedly, professionals generally assume positive credibility stereotypes because of their higher educational level and professional licensure. For example, there is a stereotype that doctors know everything about all aspects of life, not just their area of expertise. Such a stereotype would make it difficult for a client to recognize when a professional is making an error or making an unwarranted claim.

After their internships, doctors tend to not update their knowledge in areas other than their specialties, such as gastroenterology, otolaryngology, or psychiatry. However, patients are often unaware that there are variations in the accuracy or “freshness” of doctors’ medical knowledge, and they often believe that the medical instructions they hear from doctors are always correct and up to date [16]. Ultimately, professional–client epistemic teamwork becomes inflexible when a professional’s mere opinion is misunderstood as an indisputable truth, which can be detrimental to the client.

### Testimonial smothering

Since Fricker’s seminal work, additional forms of epistemic injustice have been proposed. Among these, Kristie Dotson’s [2] notion of *testimonial smothering* is illuminative in discussing the epistemic injustice frequently observed in professional–client relationships. Testimonial smothering refers to the speaker’s self-restriction of testimony in favor of content that is likely to be accurately understood by the hearer.

Dotson [2] explained testimonial smothering with an episode experienced by Cassandra Byers Harvin, a black woman who described her reluctance to engage in dialogue about race in the United States. When Harvin was asked at the public library what she was working on by a white woman, who seemed to be in her early fifties, Harvin replied that she was researching “raising black sons in this society.” The white woman immediately responded, “How is that any different from raising white sons?” Harvin sensed that the question was not innocent but derived from skepticism that she was “making something out of nothing.” Thus, instead of continuing the conversation, she made an excuse to leave — her withdrawal from the conversation being a typical example of testimonial smothering.

Dotson [2] described three mutually intertwined circumstances in which testimonial smothering occurs. The first occurs when it is perceived as unsafe for the speaker to give testimony, the second when the audience appears to lack the competence to understand the testimony accurately, and the third when the lack of competence in understanding the testimony stems from pernicious ignorance on the part of the audience.

For example, if a mere suggestion that there may be particular difficulties in raising a black son in the United States is met with immediate and severe suspicion, it may discourage people from continuing to assert it. Furthermore, the white woman Harvin met at the public library seemed to lack sensitivity to differences in child-rearing experiences that parents from different ethnic backgrounds may have, and her lack of sensitivity was related to her ignorance stemming from her membership among the ethnic majority. In such a situation, the speaker refrains from giving a voice to matters that the audience seems to lack the competence to understand appropriately.

While the lifelong dedication of professionals to their work and strong cohesion with their peers through the organization of professional associations can help maintain the quality of services, it can also lead to professional silos of ignorance and insensitivity — apparent or actual — regarding situations outside their field of expertise. Therefore, testimonial smothering in the professional–client relationship is likely to occur, not in the professional’s central area of expertise, but in peripheral fields adjacent to it. There may be a close relationship between being a minority in various attributes and mental health problems. However, if a psychiatrist belongs to an affluent social group, is part of an ethnic majority, and is a cis-gender heterosexual, the client might perceive the psychiatrist as lacking sensitivity to issues specific to underprivileged social groups or cultural and sexual minorities, unless she actively gestures otherwise. In support of this, it is known that in the United States, African American patients rated their medical visits as significantly less participatory than whites, but feel that their visits were more participatory when they saw a physician of the same race than when they saw a physician of a different race [17].

For example, assume that a depressed patient, who is also gay, had problems specific to being a sexual minority, such as having his sexual orientation outed by a friend. If the patient finds his psychiatrist is utterly ignorant of the problems common to sexual minorities, he may be discouraged from confiding important information about his problem to the psychiatrist. However, the psychiatrist would be missing out on clinically important information because the worries associated with being a sexual minority can have a significant impact on the patient’s mood states.

In summary, testimonial smothering in a professional–client relationship occurs when the client refrains from disclosing important confidential information to the professional because the professional appears to be ignorant or lacking competence to understand the information appropriately, with the client thinking, “There is no point in telling her,” or “If I say this, I might offend her.”

### Participant injustice

Christopher Hookway [3] pointed out that epistemic activities comprise not only the transmission of information but also its discussion, inquiry, and deliberation. These - along with giving testimonies, asking questions, making suggestions, and producing counterexamples - are essential components of epistemic endeavors. He showed that identity prejudice prevents some people from participating in the latter kinds of epistemic activities, which he argued is a form of epistemic injustice.

Hookway conceived a case in which a teacher discusses a philosophical subject with her pupil. The teacher is willing to accept the pupil’s testimony and provide an explanation when the pupil asks for information or complains that the textbook is difficult to understand. However, when the pupil asks a question intended to deepen their understanding of a philosophical question, rather than simply requesting information, the teacher fails to grasp the intention and mistakes the question for a mere request for information. In this case, the pupil is not a victim of testimonial injustice. However, the pupil is not treated as a true participant in the discussion. If such treatment is based on the teacher’s prejudice against the pupil, then the pupil is a victim of a kind of epistemic injustice, which Hookway called “participant injustice” [3].

Those who suffer from participant injustice, even if they attend to some epistemic activity, are considered unable to perform speech acts relevant to the activity. Fricker defined testimonial injustice as an unjust underestimation of the *credibility* of a speaker based on prejudice against their social attribute. Participant injustice arises from unjustly underestimating a speaker’s *sense of relevance* in the epistemic activity that the speaker is attending.^3^

The notion of relevance is vital when examining a professional–client relationship. A professional does not often dismiss her clients’ testimonies as untrue, although it is common to dismiss them as irrelevant. Professionals almost reflexively make judgments about the importance and relevance of information brought in by clients, saying (for example), “What he is talking about is medically/legally irrelevant.” It is important to note that professionals do not judge relevance in general, but rather relevance specific to their area of expertise.

Selecting relevant information is essential for professionals to solve clients’ problems. For example, based on fieldwork, Tanya Luhrmann [18] found that novice psychiatric residents spent much time interviewing patients and writing lengthy summaries. As they advanced in training, their interviews become shorter and their summaries more concise. To become a competent professional, one must acquire the skills to gather relevant information from the client’s words efficiently.

To gather information efficiently, professionals sometimes use questionnaires that allow just predetermined responses or closed questions, such as “How long have you had a fever?” or “Do you rent or own your house?” Closed questions allow the client to talk about just those aspects that the professional is interested in. With open questions, the client can say what he wants. However, professionals tend to focus only on what they consider relevant from a professional perspective among what the client replies.

A professional tends to retain a dominant position in communication with a client by monopolizing the judgment of relevance. The monopoly can be the result of professionals’ higher educational levels as well as their exclusive dedication to their area of expertise, which is also exacerbated by closed communication with their peers. The client may be trusted as an informant. However, he is kept in a peripheral position in epistemic teamwork. Just as the sensory and motor organs are in the nervous system, the client’s role is to report facts and adhere to the professional’s instructions, while the professional occupies the role of the central nervous system; that is, to inquire, deliberate, and make judgments.

In epistemic teamwork, it is not particularly unjust for a person to be designated only a peripheral role if that is their assigned role. For example, if an army scout discovers an enemy in the woods, the sole responsibility of the scout is to report it to his superiors as soon as possible. It is the commander’s job to select and integrate the information gathered from various sources and to develop a military plan.

However, in a professional–client relationship, it is inappropriate for the professional to behave like a commander and for the client to be treated like a scout. In this relationship, the primary goal is to solve the client’s problems, and the client should be in the commander’s seat. When considering that the information provided by the client is used to solve their problems, it is evident that marginalizing the client in epistemic teamwork could amount to epistemic injustice.

The assumption that the client is a *mere informant* and cannot perform speech acts relevant to solving the problem could demoralize the client. It might cause the client to refrain from revealing vital information, with the thought that “this information must be irrelevant.”

More importantly, the client’s complaints are usually wider than the consulted professional’s area of expertise. For example, the problems of a person who visits a lawyer for advice about an inheritance may be partially conceptualized using legal terminology, but it is likely to contain a residue that cannot be captured by legal concepts, such as problems of family discord. Therefore, when a professional tries to fit a client’s problem into a pre-acquired formula, something — personal, existential, or phenomenological — is always dropped from its scope.

To reframe the situation in terms of relevance, there is a discrepancy between what is relevant to the client’s initial concerns and what is relevant from a professional perspective. The problems of a client who consults a doctor are not entirely medical, and the problems of a client who consults a psychologist are not entirely psychological. Because of this discrepancy, some statements that are dismissed as “irrelevant” from a professional point of view may be highly relevant to the client’s initial concern.

Consider an old man with an anxiety disorder who has been visiting a psychiatrist and has been on medication for a long time. He complained to her that his anxiety was not getting any worse, but it was still there. The psychiatrist then checked the patient’s appetite, sleep, and other aspects of his health status. When she asked him how often he went out, he told her that he was so anxious about going outside that he had to ask his son, who lived with him, to drive him whenever he went out, and he confided that he had asked his son to bring him to this visit as well. When she finally asked the old man if there was anything he wanted to discuss with her, he asked, “should I still stay on the meds?” She replied that stopping the medication might make his anxiety even worse, but he could try it if he wanted to. The old man said he would continue the medication and left the office.

In this case, the psychiatrist took the patient’s question, “should I still stay on the meds?” literally and gave a medically correct answer. However, considering that the patient asked this question just after stating that he needed his son’s help to visit the clinic, what he really meant was that he would continue to cause inconvenience for his son if he continued to take medication. If the psychiatrist had been able to understand the patient’s living situation a step deeper, she would have been able to discuss the issues that were more relevant to him, such as whether the intervals between visits should be widened, or whether he was being maltreated by his son.

Hookway [3, p. 158] suggested that participant injustice can affect the implicature of speaking but did not elaborate on this topic. Therefore, this paper extends his idea and explains how a disagreement in the judgment of relevance can lead to an oversight of the implicature of the client’s utterance.

Implicature is a pragmatic phenomenon that was first systematically studied by Paul Grice [19]. It is an implicit message that is not part of what is literally said or logically entailed by the statement, but which the hearer infers as the speaker’s intention to convey from the assumption that the statement is in accordance with the four maxims of conversation (informative, truthful, relevant, and clear). Dan Sperber and Deirdre Wilson [20] integrated Grice’s four maxims of conversation into the relevance theory of communication. In this theory, an implicature is an implicit message derived from the assumption that the speaker has said something relevant to the situation in which the conversation takes place. For example, if a woman who went for dinner with a man was asked, “Did you have any trouble with him at dinner?” it might convey the implicature that the man was potentially dangerous.

Returning to the above case, the anxiety disorder patient’s question, “Should I still stay on the meds?” implicated that he was concerned about continuing to be an inconvenience for his son. However, if the professional fails to recognize the relevance from his viewpoint, she would interpret his statement only literally, and overlook the significant implicature.

## Epistemic injustice in the doctor–patient relationship

### Characteristic features of a doctor–patient relationship

A doctor is a professional with the dual role of being a provider of skills and a consultant. A doctor’s primary job is to cure illnesses and injuries. For illnesses and injuries that can be cured quickly, there is little need for epistemic collaboration between a doctor and a patient. For example, when an orthopedist reduces a dislocated shoulder, she is merely a provider of skills, like a carpenter.

Epistemic collaboration plays a vital role in the doctor–patient relationship when the patient is suffering from a chronic illness or an illness that cannot be easily cured. In treating a chronic illness, it is necessary for the doctor and the patient to share their knowledge and values and to form a consensus about which aspects to prioritize in the treatment process.

However, patients with chronic or severe illnesses are often stereotyped as weakened by their illnesses, prone to unstable emotional reactions, and unable to make calm and rational decisions [6]. When these stereotypes reach a level that can be described as unjust prejudice against the chronically ill, it can result in testimonial injustice against them.

Fricker distinguished between incidental and systematic cases of epistemic injustice. In the former, epistemic injustice occurs only in some specific situation. In the latter, epistemic injustice threatens the person’s broad range of living for a long period. The relationship between a client and a professional other than doctors is usually temporary, and the clients do not consider being a client (e.g., undergoing arbitrated divorce) to be part of their identity. For this reason, epistemic injustice in the professional–client relationship, such as those described in the previous section, is typically incidental. On the contrary, chronic illnesses, including most mental disorders, afflict patients for an extended period, and their relationships with health professionals dominate their lives because the illness interferes with their daily living. Therefore, epistemic injustice against patients with chronic illnesses — especially with mental illnesses — if it occurs, can become systematic.

However, the uniqueness of the doctor–patient relationship expands beyond the fact that there can be identity prejudice against patients. The doctor–patient relationship is a type of professional–client relationship, and a unique feature is that the problems dealt with in the relationship are related to the client’s body. To examine the implication of this feature in-depth, I refer to Fricker’s notion of objectification in the following subsection.

### Informant and source of information

Fricker [1] cited Edward Craig [21] to explain the wrongs caused by testimonial injustice. Craig [21] proposed a distinction between an *informant* and a *source of information*. An informant is a person who gives information needed by the inquirer. By contrast, source of information is a state of affairs that have some evidential value, from which people can acquire information. For example, it is possible to determine which way is north from the position of Polaris. In this case, Polaris is used as a source of information. By contrast, when asking for directions, and a passerby replies, “North is this way,” the passerby exists as an informant. Different from a source of information, the informant shares the epistemic goal of pursuing truth with the hearer, and if they share a language, the hearer can obtain information even if they have no other knowledge. A natural object can only be a source of information, but a human being can be both an informant and a source of information. For example, if one observes a man entering one’s house with a wet umbrella, one can deduce that it is raining outside. In this case, the man was treated as a source of information.

Craig [21] utilized this distinction to analyze the notion of knowledge. He considered that the concept of informant is at the foundation of the concept of knowledge because only a person who possesses knowledge can be an informant. In addition, he argued that incorporating a person into the teamwork of epistemic practice means treating that person as an informant.

Fricker [1] considered that Craig’s idea echoed Kant’s categorical imperative of treating a person not as a mere means but as an end. In other words, in order to regard a person as a full-fledged rational being, it is necessary to treat the person not only as a source of information but also as an informant. Accordingly, a person treated as a mere source of information is objectified and not treated as an epistemic subject. She maintained that the sufferers of testimonial injustice were victims of objectification, as their credibility was unjustly deflated.

However, several authors have raised objections to Fricker’s interpretation. Gaile Pohlhaus [22] argued that the victims of testimonial injustice are not deprived of their epistemic subjectivity and turned into objects but are rather treated as truncated subjects. However, the problem is that the testimony of truncated subjects is accepted only insofar as it conforms to the worldview of those in a dominant position. Their original claims are immediately rejected if they are not consistent with the dominant worldview.

Katherine Hawley [23] emphasized the difference between the trustworthiness of people and the reliability of things. She maintained that testimonial injustice is a phenomenon of distrusting a speaker who should be trusted, while the objectification of a speaker is a phenomenon of treating them - who should be treated as a human informant - as a thing that contains some information. She denoted that Fricker’s equation that testimonial injustice is the objectification of the speaker comes from conflating the two phenomena.

Accordingly, testimonial injustice and objectification are conceptually independent. In other words, most cases of testimonial injustice are not those of objectification, and there are cases of objectification that do not fall under the category of testimonial injustice, as will be discussed in the fourth section below. Therefore, it is misleading to use the concepts of “source of information” and “objectification” when discussing epistemic injustice in general. However, these notions are optimal for conceptualizing the epistemic injustice prevalent in doctor–patient relationships, because the subject matter of medicine is a patient’s sick body.

When making a medical decision, it is essential to obtain objective information from the patient through a physical examination and laboratory tests, in addition to a medical interview and history taking. As a responder to a medical interview, the patient is an *informant*, whereas as a recipient of a physical examination and laboratory tests, the patient is *a source of information*. Given this correspondence, Fricker’s thesis, “treat a person as an informant and not merely as a source of information,” corresponds to the medical maxim, “Do not just treat the organs but treat the patient as a person.”

### Objective findings and subjective symptoms

One problem in the doctor–patient relationship in contemporary medicine, however, is that patients as a source of information are becoming increasingly prioritized, and they are becoming increasingly disregarded as informants [24].

A doctor acquires objective findings regarding the patient as a source of information. Objective findings include physical signs found in a physical examination, such as jaundice and wheezing; vital signs, such as blood pressure and body temperature; results of blood tests and various imaging studies; and microscopic findings of the cells and tissues taken from the patient’s body. Objective signs detected by physical examination, such as fever, changes in pulse rate, skin swelling, and discoloration, have been vital indicators since the dawn of medicine. With the development of medical science, it has gradually become clear that organ abnormalities exist behind clinical syndromes. As organic pathology became the essence of disease, objective findings became even more emphasized. In addition, the development of various blood tests, imaging techniques, and physiological studies made it possible to detect diseases more accurately and at an earlier stage. In addition, physical interventions - such as pharmacotherapy and surgical interventions - are based on these objective findings.

However, the progress of medicine has marginalized the patient as a person in the doctor–patient relationship [25]. In earlier days, when laboratory techniques were scarce, the patient’s testimonies were often the only information available to reach a diagnosis. As laboratory techniques developed, however, the patient’s “subjective” complaints came to be regarded as less credible than objective findings.

For example, in the days before the development of upper gastrointestinal endoscopy, gastric ulcers were diagnosed based on the symptoms reported by patients, and a patient who complained of epigastric pain was never considered as not having a genuine illness. However, with the development of upper gastrointestinal endoscopy and the association of epigastric pain with inflammation and ulceration of the gastric mucosa, complaints of epigastric pain without abnormal findings in the stomach became considered as “indefinite complaints” and regarded as conditions less significant than gastric ulcers [26].

The existence of objective findings is often decisive evidence for a medical diagnosis. There are occasions when a physician must deny the veracity of a patient’s testimony based on an objective finding. For example, a man who denies being sexually active may test positive for gonorrhea, a sexually transmitted disease. There are also situations in which doctors need to proceed urgently with treatment based on a specific objective finding. For example, if an abdominal computed tomography image of a patient complaining of right lower abdominal pain shows early signs of appendicitis, the treatment is strongly recommended even if the patient says, “I am fine.” In these cases, the subservience of the patient’s testimony to the objective findings is not the result of unjustly deflating the patient’s credibility. Instead, the emphasis on objective evidence reflects an empirical attitude.

By contrast, denying the truthfulness of a patient’s testimony or downplaying its significance based on the *absence* of objective findings is highly problematic. If the disease is life-threatening or progressive, objective findings should become detectable at some point. Therefore, the lack of objective findings indicates that the patient is not in an immediately fatal condition. However, the possibility of immediate fatality of the patient is only a fraction of what is vital to the patient.

At this point, it is instructive to recall the distinction between *disease* and *illness* proposed by Arthur Kleinman [27]. “Illness” is a concept that includes the entire spectrum of the experiences of being ill for the patient. It consists of abnormal sensations, such as pain; emotions, such as anxiety about an uncertain future; behavioral changes, such as changing one’s diet and taking a long rest to cope with illness; shifts in interpersonal relationships, such as conflicts with people around the patient; and economic hardship. It is influenced by the patient’s cultural background and includes a self-understanding of the meaning of hardship in the patient’s life. By contrast, “disease” is a rephrasing of illness as an abnormality in anatomy or biological function based on the latest medical theory.

When doctors focus on the disease based on objective findings, the psychological, social, cultural, and existential dimensions of the illness experience are dismissed. In this way, miscommunication between the doctor and patient arises, because it is most important for the patient that his illness experience is understood, and his suffering and inconvenience are alleviated.

The adverse effects of the medical profession’s emphasis on objective findings are most profound in the case of diseases for which there are no objective findings or where there is little change in objective findings over time, that is, for chronic illnesses. An extreme example is an episode when curare, a muscle relaxant, was misused as an anesthetic, because doctors had long ignored patients’ testimony that they experienced severe pain as curare effectively kept them silent during surgical operations [6]. Even today, doctors are likely to doubt or downplay the validity of such illnesses as chronic fatigue syndrome and fibromyalgia, which are defined by reported symptoms such as pain and fatigue and are poorly supported by objective findings [28, 29]. Nevertheless, such disregard of reported symptoms is not justifiable from the perspective of empiricism on which medicine relies, because *absence of evidence is not evidence of absence of illness*.

When a doctor concentrates only on objective findings and denies the truthfulness or importance of a patient’s reported symptoms that are not supported by objective findings, she treats him as a mere source of information and neglects his role as an informant. Such treatment is the objectification of the patient in the truest sense of the word. At the same time, it is also an instance of testimonial injustice in that it depreciates the patient as a knower. Furthermore, the objectification of patients is accompanied by the secondary harm of deteriorating trust between doctors and patients, resulting in a decline in the quality of medical care.

## Epistemic injustice in the psychiatrist–psychiatric patient relationship

### Characteristic features of the psychiatrist–psychiatric patient relationship

A psychiatrist–psychiatric patient relationship is a type of professional–client relationship that is also a type of doctor–patient relationship. Therefore, problems that can arise in the professional–client relationship and the doctor–patient relationship can also arise in the psychiatrist–psychiatric patient relationship. In addition, the fact that mental disorders are illnesses that can undermine patients’ rationality and status as knowers can add a distinctive challenge to the psychiatrist–psychiatric patient relationship.

A characteristic of psychiatry is that it regards a patient’s statements as manifestations of illness. Of course, such a perspective is not always necessary in psychiatry. In treating mild anxiety disorders and depression, it may be possible to accept the patient’s complaints as genuine testimony and treat the patient in accordance. However, if a patient lacks insight into their illness, as is typical of patients with delusions, the patient’s behavior, including utterances, may have to be considered a part of the symptoms of mental disorder. In such cases, objectification occurs at a deeper level in psychiatry than in other medical fields, because not only objective findings obtained from the patient’s body are prioritized, but also the patient’s behaviors, including his utterances, are treated as objective findings.

As an example, consider the case of John (based on a mixture of actual cases), who was a 21-year-old earnest university student. For the past six months, he had been unable to concentrate on the lectures and his grades had started to drop. He gradually began to think that people around him were watching him. It was when he began to withdraw into his room and stopped taking showers that his family took him to see a psychiatrist.

In the consultation room, John complained strenuously to the psychiatrist that the government was being taken over by a shadowy organization and showed her a piece of paper with an organizational chart of the organization he had written. He complained that he could hear many spies around him, watching him, and discussing things that only he should know about. Based on the history of present illness, the psychiatrist considered that John had a thought disorder and impaired ability for self-care. She also judged from his utterances that he had auditory hallucinations and delusions, and informed John that he was undergoing a first episode of psychosis and probably had schizophrenia. When she suggested that he start taking medication, John reluctantly agreed, although he showed signs of disbelief toward her. A few weeks after the initiation of medication, John’s face had brightened, and he was able to go out by himself. If asked, he still admitted that a shadowy organization was taking over the government, but he was no longer actively insisting on it. As for the spies, he stated that their number had decreased. Based on these statements, she concluded that the pharmacotherapy was effective.

In this case, the psychiatrist did not take John’s statements that the government was being taken over by a shadowy organization or that spies are discussing him as genuine testimony but considered them manifestations of hallucinations and delusions and treated them as a basis for making a diagnosis of schizophrenia. The psychiatrist also treated the fact that the patient no longer actively made similar statements after the initiation of pharmacotherapy as an indicator of the effectiveness of the medication.

Similar attitudes are common in psychiatry. For instance, a dementia patient’s complaint that a neighbor has stolen her cosmetics is regarded as a behavioral and psychological symptom of dementia, owing to her memory impairment. Aggressive statements of a patient with bipolar disorder are treated as a sign of the recurrence of a manic episode. Persistent complaints of abdominal pain and worry about internal diseases are taken as a symptom of hypochondria.

In everyday conversation, people read paralinguistic signs in a each other’s testimonies and use them as sources of information. For example, when a woman says, “The man I met on the street was very tall,” with a stiff face and a trembling voice, it can be surmised that she is frightened. In such a case, the speaker’s utterances are accepted as genuine testimony, and the paralinguistic signs are considered to provide additional information. However, in the examples of psychiatric practice described above, the speakers’ utterances are not taken as constative statements about the state of an affair but are understood exclusively with respect to their mental disorders. Therefore, utterances are used as a *mere source of information* and can be said to be objectified. The following subsections discuss whether the objectification of utterance constitutes a form of epistemic injustice.

### Is objectification of utterance a form of epistemic injustice?

In the novel *To Kill a Mockingbird*, in a rural area of Alabama in the 1930s, Robinson, a black man accused of raping a white woman, pleaded his innocence in court, but the white jurors looked at him with distrust and ridicule [30]. Fricker [1] referred to this scene as a typical example of testimonial injustice.

By contrast, objectification of utterance involves a shift from evaluating utterances on a trustworthy–untrustworthy axis to evaluating them on a reliable–unreliable axis, as proposed by Hawley [23]. The objectification of utterance is one of the basic strategies to take what Peter Strawson [31] called an *objective attitude* toward psychiatric patients. Strawson maintained that in ordinary human relationships, people expect others to show goodwill and respect toward them. They naturally develop *reactive attitudes*, such as resentment, when another party shows contempt or ill will toward them; while they feel gratitude when another party shows them goodwill or kindness. Conversely, when the interlocutor is a small child or a person with severe mental disorder, who lacks the capacity to participate in mature interpersonal relations, they attempt to suppress reactive attitudes, such as resentment or gratitude. He argued that the recipient of such an attitude is not considered a member of our moral community, but an object of treatment, supervision, and correction.

Adopting an objective attitude is indispensable in the therapeutic relationship in psychiatry because the reactive attitudes that naturally arise in response to the words and deeds of patients whose rationality is impaired are often injurious to people around them. To distrust a person’s testimony or to feel resentment upon finding that the testimony was untruthful is to take a reactive attitude toward the person. Conversely, if the psychiatrist does not accept the person’s utterance as testimony but instead regards it as a symptom of illness, she can then suppress her natural emotions that are potentially injurious to the patient.

Furthermore, utterances that are regarded as symptoms of illness are not simply ignored but are incorporated into clinical reasoning as a source of information about the patient’s state of illness. In other words, in the psychiatrist–psychiatric patient relationship, the objectified utterances, instead of being accepted as constative statements, are considered reliable indicators of the patient’s condition, and are utilized in treatment planning. The objectification of utterance is invasive for the patient because it implies not taking the patient’s claims at face value. However, the present thesis argues that an instance of objectification of utterance is justified if it satisfies the following three conditions.

The first condition is that the objectification of the utterance is based on sufficient objective grounds. Fricker [1] maintained that it is a case of testimonial injustice to discount the credibility of a speaker based on the prejudice against the speaker’s social identity. Likewise, uniformly objectifying the utterances of patients with schizophrenia or dementia, solely because they have severe mental disorders, would be no different than cases of testimonial injustice. By contrast, if there is independent and sound evidence, it would not necessarily be a case of testimonial injustice to consider a patient’s utterance as a symptom of illness. This requirement resonates with Bueter’s [32] proposal that it does not constitute epistemic injustice to attribute less credibility to a patient based on “local features of the patient’s particular presentation” rather than on crude categorizations such as being mentally ill or having schizophrenia.

The second condition is that the utterance is objectified for promoting the patient’s health and avoiding the potential damage for the patient’s reputation, property, or interpersonal relationships that would ensue were it accepted as a valid illocutionary act. Assertions and promises are speech acts that announce the speaker’s commitment and “bind” the speaker, and they have various effects on the audience. For example, if a patient’s aggressive assertions are taken seriously, the hearer might be hurt and develop feelings of resentment and hatred toward the patient. The patient would also be regarded as a bad person because of the unjustifiably offensive remarks. While avoiding those harms, the psychiatrist utilizes the utterance as an indicator of the patient’s mental health status and integrates it into his treatment planning.

The third condition is that the objectification of utterance is confined to the minimum extent necessary to compartmentalize the patient’s pathological behaviors rather than covering all the patient’s utterances. Strawson [31] contrasted an objective attitude with a reactive attitude and took a simplistic view that an objective attitude should be adopted for patients with severe mental disorders. However, it is rarely necessary to take an entirely objective attitude, even for people with severe mental disorders. Those who work with patients usually adopt a patchwork of reactive and objective attitudes.

In discussing stigma against mental disorders, Abigail Gosselin [33, p. 84] states:

For example, a person with anorexia may be completely irrational regarding food and weight, but be rational on all other topics; a person with schizophrenia or bipolar disorder may experience delusions of hearing messages emanating from objects yet have normal perception and reasoning ability in contexts and at times where such delusions do not arise. Over-generalizing or misunderstanding the nature and scope of impairment can result in epistemic injustice when the discrediting that occurs as a result of these judgments is unfair and not justifiable, particularly when it occurs in conjunction with stigma.

Thus, to establish a mutually respectful relationship and therapeutic alliance with the patient, the psychiatrist must make “complicated and nuanced judgments which are more accurate [33, p. 78]” regarding the credibility of the mentally ill patient.

The role of the psychiatrist is to treat disorders that partially impair the patient’s rationality and status as a knower. Therefore, the objectification of utterance is justified so long as it is minimal, is based on proper grounds, and when its benefits outweigh its harms. In such cases, it does not amount to epistemic injustice.

### Justifiable and unjustifiable cases of excessive objectification

However, psychiatrists’ objectification can be excessive. A typical example of excessive objectification of utterance is mistaking a patient’s factual statement for a delusion. Another example of excessive objectification, if not objectification of utterance, is misjudging a meaningful behavior or emotional reaction to a situation as a symptom of mental disorder. Psychiatrists are afraid of overlooking mental disorders. However, this increases the likelihood of false positives. For example, contemporary psychiatry has been criticized for misdiagnosing understandable grief that follows an experience of significant loss as a case of clinical depression [34]. Excessive objectification of emotions and behaviors other than utterances may also have an epistemically undesirable consequence of impeding dialogue about the meaning and purpose they have within the patient’s living environment, hindering the psychiatrist’s understanding of the patient and the patient’s own self-understanding. Then, is excessive objectification a kind of epistemic injustice? To consider this point, two extreme cases are contrasted below.

At one extreme is the Saks’ experience, outlined in the introduction [10]. When Saks visited the emergency room for an unusual and severe headache, she was sent home without sufficient medical checkup because the emergency doctor found that she had a history of schizophrenia and assumed that the headache complaint was part of a psychotic episode. This is an unjustifiable case of objectification of utterance because it does not satisfy the three conditions that justify the objectification of utterance.

First, the objectification of utterance experienced by Saks was not based on objective grounds. Instead, the emergency doctor interpreted her complaints as a symptom of illness based solely on the information that she had schizophrenia. Second, in this case, objectification was not done for Saks’ sake, but for the emergency doctor to avoid a time-consuming medical examination and treatment. Third, objectifying her utterance based solely on the fact that she has schizophrenia would make it impossible to establish a mutually respectful relationship and therapeutic alliance with her.

Furthermore, Saks’ case lacked psychiatric care for her utterance, which was attributed to her mental disorder. If a visit to the emergency room with a complaint of headache were itself a symptom of schizophrenia, it would be vital information suggesting a worsening of the schizophrenia, and a reconsideration of the treatment plan would be necessary. Nevertheless, the emergency doctor did nothing but send her home. Considering all these points, it seems appropriate to characterize Saks’ experience as a case of testimonial injustice based on prejudice against people with mental disorders.

Saks’ experience is an instance of what Bueter [32] has investigated under the name of diagnostic overshadowing. When the patient already has a diagnosis of mental illness, physicians are prone to misconstrue the symptoms of physical illness as being caused by the mental illness and fail to provide sufficient medical workup or appropriate treatment for the physical illness. Saks’ case is an example of diagnostic overshadowing that occurred in the emergency room, but similar errors could be committed by psychiatrists because they are known to have similar negative stereotypic image and implicit bias toward patients with mental illness as those of non-professionals [35, 36].

Nonetheless, it is epistemically challenging to distinguish utterances that are symptomatic from those that are not when a patient has delusions. For this reason, psychiatrists and other mental health professionals cannot avoid overdoing (or underdoing) the objectification of patients’ utterances to some extent.

Abdi Sanati and Michalis Kyratsous’ [37] case of Mr. M.G., a young African-Caribbean man with schizoaffective disorder, is an example of the opposite extreme. Mr. M.G. was picked by the police, following a threat to attack another person, and brought to a psychiatric hospital for further assessment. He accused the person he was threatening to attack of abusing a close relative of his. The psychiatrist who interviewed him considered his statement delusional; hence, he was involuntarily hospitalized under the Mental Health Act. However, it was later found that his relative was in fact abused by that person.

Even though it turned out to be a mistake, the psychiatrist had specific reasons to suspect that the man’s utterances were part of delusions at the outset, because he also manifested “irritable mood, some degree of pressured speech, delusions of reference from TV and was still expressing persecutory ideas with various contents [37, p. 482].” In addition, the imminent threat of violence made it impossible to take the time to acquire collateral information. Furthermore, the psychiatric team tried to offer him psychiatric treatment based on the assumption that he had a delusion that could result in violence. Therefore, it is too harsh to regard it as a case of epistemic injustice.

Fricker [1] argued that if a faulty judgment to discount the speaker’s credibility is based on a generally reliable stereotype rather than on prejudice against the group to which the speaker belongs, it should be viewed as *epistemic bad luck* rather than *epistemic injustice*, for there is nothing morally objectionable about such a misjudgment. Her examples of epistemic bad luck include a case in which a speaker is judged untrustworthy because he avoids looking the hearer in the eye and pauses in mid-sentence as if he is fabricating a story; however, the odd behavior was due to his extreme shyness. Mr. M.G.’s experience should rather be construed as a case of epistemic bad luck.

The case of Saks and that of Mr. M.G. are two extremes. Actual cases of excessive objectification will often not be readily classified into one of these categories because both prejudice and evidence seem to contribute to erroneous judgments. Psychiatrists are specialists in mental disorders and are required to make more fine-grained judgments than usual regarding the credibility of patients with mental disorders. At the same time, constraints of time and resources may compel them to make judgments based on some general stereotypes. In addition, the degree to which false negatives as well as false positives are acceptable depends also on the severity of the potential harm that would be expected were each type of error to occur. In cases like Saks’, more evidence would be needed to justify the objectification because of a greater potential harm associated with excessive objectification. Conversely, in cases like Mr. M.G.’s, less proof would justify the objectification because a greater potential harm is associated with insufficient objectification.

Indeed, the dichotomy of epistemic injustice and epistemic bad luck — or of guilty and innocent — may not fit well with what is occurring in the psychiatrist-psychiatric patient relationship. There are gradations in the justifiability of objectification. It may be more realistic to think of psychiatrists as having a general obligation to make prudential judgments regarding objectification at individual as well as organizational levels.

## Ameliorating epistemic injustice in therapeutic relationships in psychiatry

### Interim summary

The previous sections investigated epistemic injustice predisposed by psychiatrists’ roles in relation to their patients, stratifying it into three stages. A psychiatrist is also a professional and a doctor. As professionals, psychiatrists have expert knowledge of mental disorders and their treatment, and within this limited field, they know what is relevant to the disorder and treatment better than the patient. However, if this leads to the false perception that psychiatrists are infallible, if they are too ignorant of matters outside the narrowly defined field of psychiatry, or if they are unaware of the relevance of what patients say in contexts outside of psychiatry, then patients may become discouraged to say what they know or relegated to a peripheral position in the epistemic teamwork with psychiatrists. As doctors, psychiatrists are required to focus on patients’ physical abnormalities and prioritize objective findings, if there are any. However, this may lead to denial or disregard of the patient’s complaints when there are no objective findings or little change in the objective findings. Furthermore, psychiatrists must sometimes objectify a patient’s utterances as manifestations of his illness since mental disorders threaten the patient’s rationality and status as a knower. However, it is often difficult to distinguish between genuine testimonies from utterances attributable to mental disorders, leading to excessive objectification.

Based on the discussion in the previous sections, the following subsections outline what is needed to reduce epistemic injustice in the therapeutic relationship in psychiatry. Since a psychiatrist–psychiatric patient relationship is a kind of doctor–patient relationship and a kind of professional–client relationship, the proposals from each perspective are described separately.

### Proposals from the perspective of the professional–client relationship

In a professional–client relationship, there is asymmetry of knowledge in the professional’s area of expertise, and professionals take the lead in discerning credible from incredible information. This applies equally to psychiatry. However, psychiatrists must also be open to admitting mistakes. The misconception that psychiatrists cannot make mistakes leads to inflexible epistemic teamwork.

Furthermore, the psychiatrist needs to behave in a way that the patient expects her to have hermeneutical resources to understand what he is saying. If the patient cannot expect this, he may think, “There is no use in telling the doctor about this,” and testimonial smothering ensues. Psychiatrists are not omniscient and cannot understand everything outside their field of expertise. However, they should develop “peripheral vision.” In other words, they need to have a basic understanding of the areas neighboring psychiatry, such as poverty, sexual/ethnic minorities, and traumatic experiences, in addition to their expertise in the specific domain. Some of these areas are gradually gaining attention and are referred to as social determinants of mental health [38].

Psychiatrists need to select relevant information from psychiatric perspectives to solve problems brought by the patients. However, it is crucial to understand that patients may have different perspectives of relevance. When the relevance a patient has in mind is ignored, the implicature of the patient’s statement is overlooked, and the patient is dismissed as merely performing an illocutionary act that is spoken explicitly. When the psychiatrist regularly overlooks the patient’s implicatures, the patient is prevented from participating in epistemic collaboration and ultimately right healing.

Indeed, both psychiatrists and patients are equally prone to overlooking implicatures. In other words, not only do psychiatrists frequently overlook the implicatures of their patients’ statements, but so do patients also frequently overlook the implicatures of their psychiatrists’ statements. However, since the goal of the psychiatrist–psychiatric patient relationship is to solve the patient’s problems, the relevance for the patient must come first.

Fortunately, there is a sign suggesting an oversight of implicature. An oversight of implicature should be considered *whenever* the psychiatrist *feels* that a patient, whose linguistic or cognitive capacity is not significantly impaired, is saying something that is medically/psychiatrically irrelevant. It is unusual for such a patient to make a completely irrelevant statement. If it is irrelevant from a medical/psychiatric viewpoint, it is likely to be relevant from other viewpoints. Therefore, when a psychiatrist is frustrated because she feels that a patient is saying something irrelevant, it is a perfect time to reconsider whether she has overlooked something or to ask the patient about the intention of the statement.

However, this does not mean that everything relevant from the patient’s perspective should be treated in the therapeutic relationship. Psychiatrists are not omnipotent, and the further away a problem is from their area of expertise, the more difficult it is for them to navigate successfully. In some cases, the most relevant problem for the patient may be beyond the psychiatrist’s expertise. In such cases, psychiatrists should admit to the patient that they are incompetent. Therefore, even though the relevance for the patient is paramount, both parties must agree on what is to be addressed in the therapeutic relationship.

Alistair Wardrope [39] and Anita Ho [40] independently mentioned the importance of epistemic humility. Epistemic humility is an attitude of recognizing the limitations of one’s knowledge and one’s fallibility, and actively seeking support from others to compensate for these shortcomings. It is easy for psychiatrists to become arrogant because of their superior psychiatric knowledge compared to patients. However, to best address the issues that patients are concerned about, it is essential to be interested in fields adjacent to but outside of psychiatry and to attempt to know what is visible and relevant from the patient’s perspective. Psychiatrists’ work ethic of service orientation can help ameliorate epistemic injustice if it is tied to epistemic humility.

### Proposals from the perspective of the doctor–patient relationship

The doctor–patient relationship is characterized by the fact that the subject matter of the relationship is the patient’s sick body. The development of medical science has disclosed the nature of various diseases and invented various laboratory techniques. Nevertheless, it has also led to an overemphasis on patients as sources of information and a disregard of patients as informants. This situation can be appropriately described as the objectification of patients.

The problem may be relatively mild in psychiatry, because laboratory techniques are underdeveloped in psychiatry and interviewing is still the most important way to diagnose mental disorders. Accordingly, psychiatry is a specialty in medicine that depends heavily on epistemic collaboration with the patient.

However, objective findings are also crucial for psychiatry. Objective findings here do not refer to the results of laboratory tests, but rather objective signs, such as the patient’s behaviors, physiological states, and facial expressions. The systematic observation of these signs has been called a mental status examination. Psychiatrists must sometimes prioritize the findings of the mental status examination over the patient’s testimony. For example, if a depressed patient who cannot read a newspaper article because of thought inhibition and intense agitation were to insist, “I am cured of depression and can go to work tomorrow,” the psychiatrist would have to deny the request.

Psychiatrists are prone to downplay reported abnormalities that are unobservable by others more than overtly observable behavioral abnormalities. For example, in treating schizophrenic patients, increasing the dosage of antipsychotic medication is readily proposed, because people around the patient easily observe the patient’s delusional statements. On the contrary, reducing medication dosage is not readily approved, because fatigue and sexual dysfunction caused by antipsychotic medication are difficult to notice without being reported by the patient.

For the patient, being active is just as important as being free from delusions. Emphasizing the patient’s first-person perspective leads to respecting the patient as an epistemic subject. This may also lead to better therapeutic outcomes through improved adherence to the treatment.

### Proposals specific to the psychiatrist–psychiatric patient relationship

Psychiatrists sometimes need to interpret their patient’s utterances as symptoms of a mental disorder, instead of taking them at face value. To minimize excessive objectification, fine-grained judgments of credibility must be made, based on the information available from the patients themselves and those around them. In addition, postponing the judgment regarding objectification is preferable unless an urgent decision is necessary.

The objectification of utterance harbors further issues, because, even if it is warranted to do so, it challenges the speaker’s status as a knower and can damage the patient’s self-esteem. The patient might develop a sense of distrust toward the psychiatrist, which disrupts the epistemic teamwork between them. This is somewhat analogous to surgical operations. Just as it is not a crime for a surgeon to incise the patient’s body, it is not a case of epistemic injustice for a psychiatrist to objectify the patient’s utterance so long as there is appropriate justification. The surgeon’s special moral standing cannot be separated from her obligation to close the wounds she has created. Similarly, the psychiatrist must also work to mend the relationship of trust, restore the patient’s self-esteem, and ultimately recover (and restore) the patient’s epistemic subjectivity.

When the objectification of utterance is required, the patient is not considered to know fully about his own disorder or symptoms. In this context, it is important to note that the patient can become an epistemic subject even when he cannot be regarded as a *knower*. As Hookway [3] has shown, transmitting information is not the only epistemic activity; questioning, discussing, and deliberating are also cardinal epistemic activities. Therefore, it is possible to involve the patient in epistemic teamwork as an *inquirer*.

Moreover, it is beneficial for a patient to participate in epistemic teamwork as an inquirer in the therapeutic process because the subject matter of the psychiatrist–psychiatric patient relationship is the patient’s own mental disorder, and knowing about oneself can alleviate — if not cure — the mental disorder. Specifically, psychiatrists should share their clinical reasoning process with patients as much as possible, even if they have delusions. When it is unavoidable to objectify utterances, it is necessary to explain why they cannot be accepted at face value and spend sufficient time discussing them. This would demonstrate to the patient that he is a member of the epistemic teamwork even when his credibility as an informant is denied. Treating the patient as an inquirer of knowledge mitigates the anger and distrust caused by being objectified by the psychiatrist and empowers the patient in cultivating the insight into his disorder.

## Conclusion

This paper has argued that the epistemic injustice suffered by psychiatric patients in the therapeutic environment is not solely based on prejudice against mental disorders. Psychiatrists are professionals who provide treatment within the scope of their expertise, and as doctors, are required to focus on objective findings obtained from patients’ bodies. In addition, psychiatrists sometimes must take patients’ utterances as manifestations of mental disorders. These occupational roles predispose psychiatrists or mental health professionals in general, to committing epistemic injustice to psychiatric patients in the therapeutic relationship.

The issues raised in this paper are only a subset of the epistemic injustice prevalent in psychiatry. This essay did not touch on the structural problems of psychiatry, such as epistemic injustice derived from the power of psychiatrists and the constraints of time and financial resources. It also did not address the epistemic injustice experienced by psychiatric patients outside the therapeutic relationship, whereby patients have been excluded from psychiatric research and policymaking. However, the problems described in this paper are characteristic of the therapeutic relationship in psychiatry and demonstrate the need to consider different social domains separately when discussing epistemic injustice.

## Data Availability

Not applicable.

## Footnotes

1 Only a portion of previous literature on this topic is mentioned. Ian James Kidd summarizes previous publications regarding epistemic injustice in healthcare, including psychiatry. See, https://ianjameskidd.weebly.com/epistemic-injustice-and-illness-bibliography.html.

2 Some theorists do not admit that psychiatry is a medical profession. Thomas Szasz, for example, has repeatedly argued that mental illness is a myth, and that psychiatry is not a subspeciality of medicine, but a part of the system of social control [41]. Others may consider psychiatry to be more akin to clinical psychology than medicine. These points should be taken seriously. A limitation of this paper is that it focuses on psychiatry as a medical profession, and forensic or psychotherapeutic aspects of psychiatry is not adequately dealt with here.

3 Hookway considered that the notion of participant injustice and that of testimonial injustice cannot be clearly distinguished and suggested that the former is better considered as a superordinate concept that encompasses the latter.
